# The impact of order with radiation therapy in stage IIIA pathologic N2 NSCLC patients: a population-based study

**DOI:** 10.1186/s12885-020-07309-y

**Published:** 2020-08-26

**Authors:** Hongxia Duan, Long Liang, Shuanshuan Xie, Changhui Wang

**Affiliations:** Department of Respiratory Medicine, Shanghai Tenth People’s Hospital, Tongji University School of Medicine, #301, Mid Yanchang Rd, Shanghai, 200072 China

**Keywords:** Non-small-cell lung carcinoma, Survival, Radiotherapy, Surgery, SEER

## Abstract

**Background:**

The aim of this study was to investigate the optimal order of radiation therapy in patients affected by stage IIIA pathologic N2 (IIIA/N2) non-small-cell lung cancer (NSCLC) and to identify its potential risk factors.

**Methods:**

17,654 (8786 men and 8868 women) diagnosed with NSCLC stage IIIA-N2 from 2004 to 2015 patients were identified in the Surveillance, Epidemiology, and End Results (SEER) database. Among the relevant clinical parameters, we evaluated overall survival (OS), lung cancer-specific survival (LCSS) and other variables such as age, sex and tumor size in patients who were treated with different combinations of surgery and radiotherapy strategies.

**Results:**

We discovered that surgery benefit in younger IIIA/N2 NSCLC patients (age ≤ 75), and compared with surgery only, preoperative radiotherapy significantly improved the survival rate most (*p* < 0.001). When we performed the OS and LCSS analysis in the subgroup of patients’ age > 75 years old, who underwent postoperative radiotherapy (PORT) had the highest survival rate (*p* < 0.001). Multivariate analyses showed that the following parameters had a negative impact on survival: female sex, older age, no chemotherapy, large tumor size, high tumor grade, no surgery or radiotherapy.

**Conclusions:**

In IIIA/N2 NSCLC patients, surgery, radiotherapy and chemotherapy were associated with improved OS and LCSS. Younger patients underwent surgical resection and chemotherapy, the main population we studied, benefited most from preoperative radiotherapy in all orders with radiation therapy (*p* < 0.001). In patients more than 75 years old, there was no clear benefit from only surgery, and PORT was recommended in case of having surgery.

## Background

Lung cancer is the leading cause of cancer-related mortality worldwide [1]. Lung cancer includes small cell lung cancer (SCLC) and non-small cell lung cancer (NSCLC), the major type of NSCLC are adenocarcinoma (AD) and squamous cell carcinoma (SQCC). In patients diagnosed with lung cancer, 15% are stage IIIA NSCLC [2–4], while stage IIIA pathologic N2 (IIIA/N2) account for 50% of the locally advanced NSCLCs cases [5–7]. NSCLC patients in IIIA stage having a tumor size T1–T2 (T2: tumor > 3 cm and ≤ 5 cm) and M0 (without distant metastasis), along with ipsilateral mediastinal and/or subcarinal lymph node (N2), are diagnosed as IIIA/N2 NSCLC according to the 8th edition TNM Stage Classification [8]. N2 are classified into three different groups: occult N2, resectable N2, and non-resectable N2 [9]. Therefore, the optimal treatment for IIIA/N2 NSCLC is still controversial because stage IIIA/N2 NSCLC patients form a very broad and diverse population [10, 11].

Currently, surgery is still the standard treatment of early-stage NSCLC, but the 5 year survival rate is only 50 to 60% [6], with a risk of locoregional recurrence of 20–40% in node-positive patients [12]. Thus, radiotherapy and/or chemotherapy combined with surgery represent the current therapeutic options for these patients. Adjuvant chemotherapy was considered to enhance survival in IIIA NSCLC patients with surgery [13, 14], but 20–40% of patients still had a local tumor failure [12]. Thus, radiotherapy, including preoperative radiotherapy and postoperative radiotherapy (PORT) is important and necessary.

Since tumor size, lymph node involvement, and comorbidities can widely vary among patients, the idea of having a universal treatment plan for stage IIIA/N2 NSCLC patients seems not feasible. While some studies confirmed that preoperative radiotherapy significantly improve survival [15, 16], other studies showed PORT demonstrated better survival instead [17, 18]. Thus, by retrospectively studying the outcomes of IIIA/N2 NSCLC patients that underwent surgery with either pre- or post-operative radiotherapy or both, we sought to answer the question of which strategy is ideal. Since prospective clinical study are lacking, we perform a retrospective study by using data from the Surveillance, Epidemiology, and End Results (SEER) database to determine which clinical parameters have an impact in the therapy outcome and to provide clinicians and patients more information to make an informed decision.

## Methods

### Data source

We used the US National Cancer Institute’s SEER database, which contain data from 18 registered cancer institute covering nearly 26% of the total US population [19]. The database coverage which is considered an accurate statistical representation of the U.S. population affected with cancer [20]. SEER*-Stat software, version 8.3.2, was used to extract data from the database.

### Cohort

**Fig. 1:**
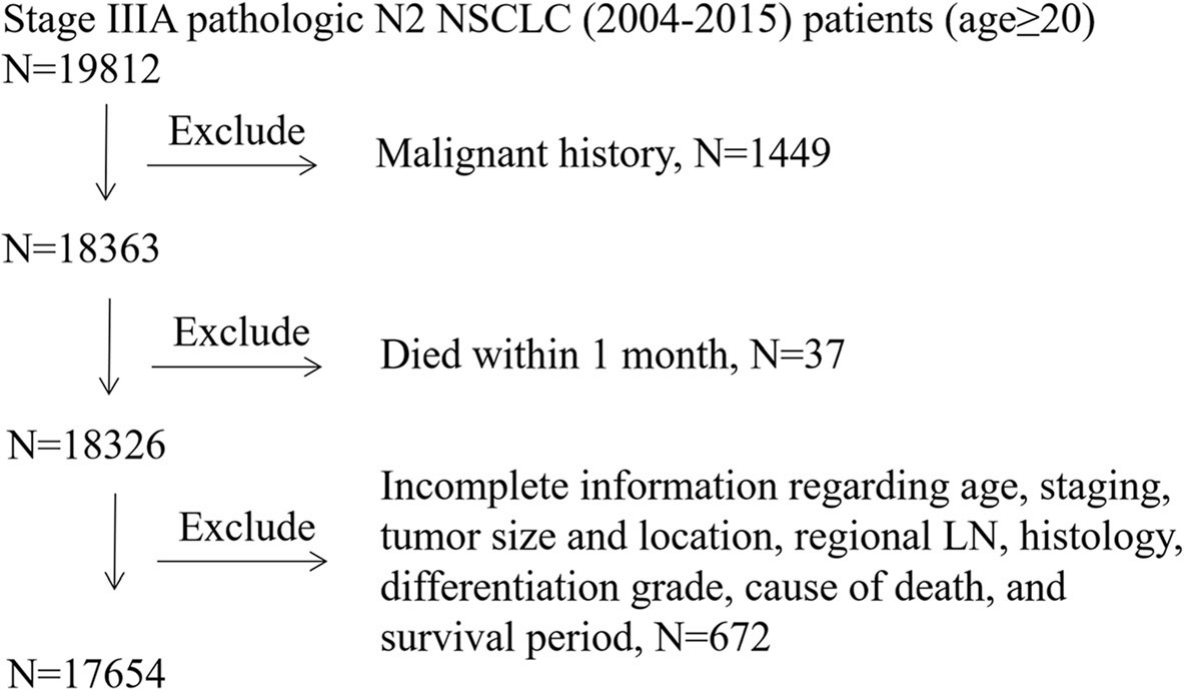
Patient selection for this study

The cohort include patients who were pathologically diagnosed with lung adenocarcinoma (AD) (histological codes 8244, 8245, 8250–8255, 8260, 8290, 8310, 8323, 8333, 8480, 8481, 8490, 8507, 8550, 8570, 8571, 8574, and 8576), squamous cell carcinoma (SQCC) (histologic codes 8052, 8070–8075, 8083, 8084, 8123), and large cell carcinoma (LCC) (histological codes 8012–8014, 8046, 8050, 8003, 8004, 8022, 8031–8035, 8082, 8200, 8240, 8249, 8430, 8560, 8562, 8980) during a 10-year period, from 2004 to 2015. Patients graded as stage T1–2 and N2 were included in this study, while those having a previous malignant disease or distant metastasis were excluded, along with patients who died within 30 days after surgery. Another exclusion criteria was the lack of complete information for the following parameters: age, complete staging, tumor size and location, regional LN examination results, histology, differentiation grade, cause of death, and survival period. Figure 1 showed the detailed case selection process.

### Covariates

Demographic parameters included age, sex, race, insurance coverage, marital status, years of diagnosis, region, education, and median household income. Tumor characteristics included size, histology, T stage (based on the Eighth Edition Lung Cancer Stage Classification), primary site, and pathologic differentiation grade and laterality.

### Statistical analysis

Pearson’s chi-square test was used to assess the baseline parameters and to evaluate the association between the groups. The Kaplan-Meier method was used to generate survival curves, the log-rank test was used to examine the differences in survival among subgroups and multivariate Cox Proportional Hazards Analysis was used to examine the effects of multiple potential prognostic factors on survival. Overall survival (OS) and lung cancer-specific survival (LCSS) were the endpoint measurements. OS was calculated from diagnosis to death from any cause, while LCSS was calculated from the time of diagnosis to death from lung cancer. OS and LCSS were estimated using follow-up data through 2017 and compared in different groups by using the Kaplan-Meier method. All tests were two-sided and *p* < 0.05 was considered to be significant. All analyses were performed using the SPSS software, version 22.0 (SPSS Inc. Chicago, IL).

## Results

### Baseline cohort characteristics

Based on the inclusion criteria, this study cohort was formed by 17,654 IIIA/N2 NSCLC patients, of which 8786 males and 8868 females. Among the patients, 5512 (31.22%) were treated neither with surgery or radiotherapy, 7184 (40.69%) received surgery only, 652 (3.69%) were given preoperative radiotherapy, 4206 (23.82%) were given PORT, and 100 (0.57%) were treated with radiotherapy both before and after surgery. The demographic and clinical parameter of patients are listed in Table 1. The tumor size and age were discretized and segmentation points were generated according to TNM stages (T1: tumor ≤3 cm T2: tumor > 3 cm and ≤ 5 cm) and by Youden index maximization, respectively.

**Table 1:** Demographic and Clinical Parameters of patients with IIIA/N2 NSCLC

Characteristics	No surgery or Radiotherapy (*n* = 5512)	Surgery only (*n* = 7184)	Preoperative radiotherapy (*n* = 652)	Postoperative radiotherapy (PORT, *n* = 4206)	Radiotherapy both before and after surgery(*n* = 100)	*P* value for X^2^
**Gender**						<0.001
Male	2572(46.66%)	3680(51.22%)	329(50.46%)	2152(51.17%)	53(53.00%)	
Female	2940(53.34%)	3504(48.78%)	323(49.54%)	2054(48.83%)	47(47.00%)	
**Age**						<0.001
< =75	3893(70.63%)	4940(68.76%)	608(93.25%)	3567(84.81%)	92(92.00%)	
> 75	1619(29.37%)	2244(31.24%)	44(6.75%)	639(15.19%)	8(8.00%)	
**Histologic type**						<0.001
Adenocarcinoma	2081(37.75%)	3831(53.33%)	342(52.45%)	2486(59.11%)	65(65.00%)	
Squamous	2118(38.43%)	1922(26.75%)	173(26.53%)	1003(23.85%)	19(19.00%)	
Adenosquamous	67(1.22%)	158(2.20%)	17(2.61%)	88(2.09%)	1(1.00%)	
Large cell	144(2.61%)	201(2.80%)	23(3.53%)	129(3.07%)	1(1.00%)	
OTHER	1102(19.99%)	1072(14.92%)	97(14.88%)	500(11.89%)	14(14.00%)	
**Tumor Size (cm)**						<0.001
≤ 3	2318(42.05%)	3765(52.41%)	295(45.25%)	2483(59.03%)	47(47.00%)	
3–5	3194(57.95%)	3419(47.59%)	357(54.85%)	1723(40.97%)	53(53.00%)	
**Race**						<0.001
White	4444(80.62%)	5810(80.87%)	544(83.44%)	3436(81.69%)	78(78.00%)	
Black	763(13.84%)	802(11.16%)	68(10.43%)	482(11.46%)	9(9.00%)	
Others	297(5.39%)	553(7.70%)	40(6.13%)	281(6.68%)	13(13.00%)	
Unknown	8(0.15%)	19(0.27%)	0(0.00%)	7(0.17%)	0(0.00%)	
**Primary Site**						<0.001
Upper lobe	3454(62.66%)	4302(59.88%)	447(68.56%)	2628(62.48%)	65(65.00%)	
Middle lobe	241(4.37%)	326(4.54%)	34(5.21%)	226(5.37%)	5(5.00%)	
Lower lobe	1337(24.26%)	2141(29.80%)	138(21.17%)	1098(26.11%)	27(27.00%)	
NOS	155(2.81%)	195(2.71%)	9(1.38%)	123(2.92%)	0(0.00%)	
Overlapping lesion	33(0.60%)	64(0.89%)	6(0.92%)	28(0.67%)	2(2.00%)	
Main bronchus	292(5.30%)	156(2.17%)	18(2.76%)	103(2.45%)	1(1.00%)	
**Grade**						<0.001
Grade I	154(2.79%)	327(4.55%)	23(3.53%)	138(3.28%)	3(3.00%)	
Grade II	900(16.33%)	1935(26.93%)	138(21.17%)	1021(24.27%)	26(26.00%)	
Grade III	1722(31.24%)	2427(33.78%)	278(42.64%)	1361(32.36%)	39(39.00%)	
Grade IV	83(1.51%)	122(1.70%)	5(0.77%)	62(1.47%)	3(3.00%)	
unknown	2653(48.13%)	2373(33.03%)	208(31.90%)	1624(38.61%)	29(29.00%)	
**Laterality**						0.001
Right-origin of primary	3421(62.06%)	4316(60.08%)	405(62.12%)	2631(62.55%)	67(67.00%)	
Left-origin of primary	2063(37.43%)	2840(39.53%)	246(37.73%)	1531(36.40%)	33(33.00%)	
Paired sit	23(0.42%)	20(0.28%)	1(0.15%)	38(0.90%)	0(0.00%)	
Only one side - side unspecified	4(0.07%)	6(0.08%)	0(0.00%)	4(0.10%)	0(0.00%)	
Not a paired site	1(0.02%)	2(0.03%)	0(0.00%)	2(0.05%)	0(0.00%)	
**Insurance status**						<0.001
Medicaid	594(10.78%)	768(10.70%)	45(6.90%)	399(9.49%)	7(7.00%)	
Uninsured	101(1.83%)	118(1.65%)	10(1.53%)	59(1.40%)	2(2.00%)	
Unknown	1316(23.88%)	1858(25.86%)	180(27.61%)	847(20.14%)	29(29.00%)	
Insured	3501(63.52)	4440(61.80%)	417(63.96%)	2901(68.97%)	62(62.00%)	
**Marital status**						<0.001
Married	2812(51.02%)	3644(50.72%)	393(60.28%)	2440(58.01%)	58(58.00%)	
Single	671(12.17%)	887(12.35%)	76(11.66%)	467(11.10%)	12(12.00%)	
Divorced	681(12.35%)	903(12.57%)	93(14.26%)	540(12.84%)	12(12.00%)	
widowed	1079(19.6%)	1401(19.50%)	63(9.66%)	574(13.65%)	11(11.00%)	
Unknown	181(3.28%)	262(3.65%)	20(3.07%)	131(3.11%)	1(1.00%)	
Unmarried or domestic partner	6(0.11%)	12(0.17%)	1(0.15%)	12(0.29%)	0(0.00%)	
Separated	82(1.49%)	75(1.04%)	6(0.92%)	42(1.00%)	6(6.00%)	
**Year of diagnosis**						<0.001
2004–2007	1756(31.86%)	2399(33.39%)	241(36.96%)	1105(26.27%)	37(37.00%)	
2008–2011	1982(35.96%)	2437(33.92%)	216(33.13%)	1326(31.53%)	35(35.00%)	
2012–2017	1774(32.18%)	2348(32.68%)	195(29.91%)	1775(42.20%)	28(28.00%)	
**Region**						<0.001
EAST	2733(49.58%)	3243(45.14%)	283(43.40%)	2148(51.07%)	38(38.00%)	
NORTHWEST	1965(35.65%)	3082(42.90%)	265(40.64%)	1417(33.69%)	50(50.00%)	
SOUTHWEST	661(11.99%)	647(9.01%)	86(13.19%)	561(13.34%)	10(10.00%)	
Pacific Coast	153(2.78%)	212(2.95%)	18(2.76%)	80(1.90%)	2(2.00%)	
**High school education**						<0.001
≥ 21	977(17.72%)	1524(21.21%)	102(15.64%)	590(14.03%)	19(19.00%)	
13–20	1823(33.07%)	2293(31.92%)	159(24.39%)	1199(28.51%)	32(32.00%)	
7–12.99	2363(42.87%)	2968(41.31%)	344(52.76%)	2101(49.95%)	41(41.00%)	
< 7	349(6.33%)	399(5.55%)	47(7.21%)	316(7.51%)	8(8.00%)	
**Median household income, in tens**						<0.001
< 38,000	422(7.66%)	570(7.927%)	28(4.29%)	290(6.89%)	5(5.00%)	
38,000–47,999	1080(19.59%)	1097(15.27%)	88(13.50%)	731(17.38%)	14(14.00%)	
48,000–62,999	2064(37.45%)	2842(39.56%)	276(42.33%)	1588(37.76%)	40(40.00%)	
> 63,000	1946(35.30%)	2675(37.24%)	260(39.88%)	1597(37.97%)	41(41.00%)	
**Chemotherapy**						<0.001
Yes	4221(76.58%)	3184(44.32%)	631(96.78%)	3791(90.13%)	96(96.00%)	
no	1291(23.42%)	4000(55.68%)	21(3.22%)	415(9.87%)	4(4.00%)	

Abbreviations: *NSCLC* Non-small cell lung cancer, *IIIA/N2* Stage IIIA pathologic N2

**Fig. 2:**
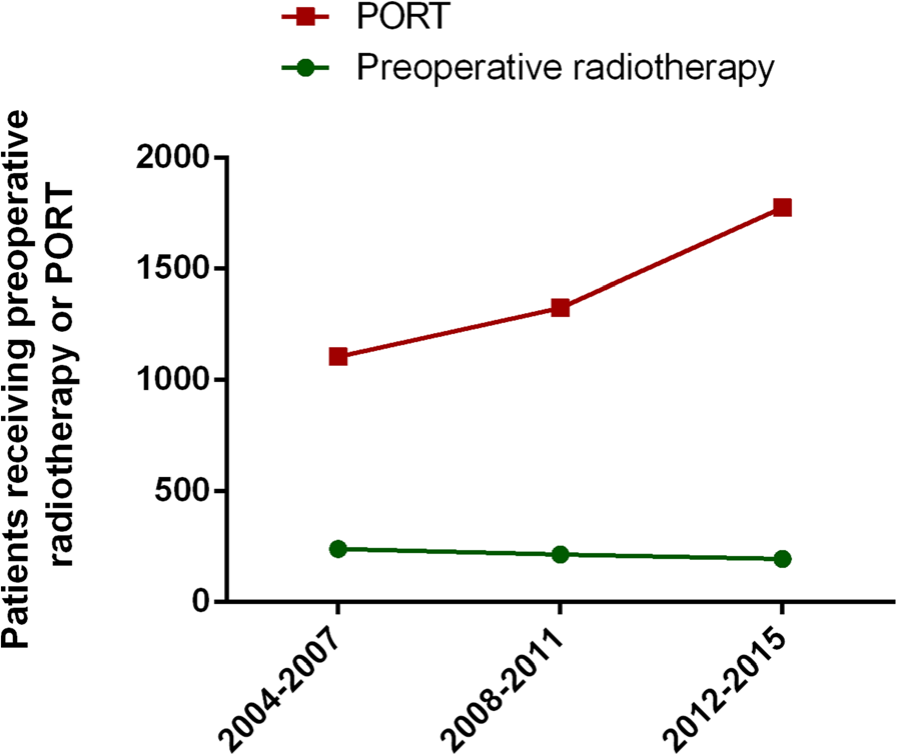
Number of IIIA/N2 NSCLC patients underwent preoperative radiotherapy and PORT from 2004 to 2015. There were 241, 216, 195 IIIA/N2 NSCLC patients received preoperative radiotherapy with surgery in 2004–2007, 2008–2011 and 2012–2017, respectively. Whereas, the number of IIIA/N2 NSCLC patients who underwent PORT with surgery were 1105, 1326, 1775 in the same time periods as above. Abbreviations: NSCLC, non-small cell lung cancer; IIIA/N2, stage IIIA pathologic N2; PORT, postoperative radiotherapy

There were statistically significant differences in all the baseline parameters between groups (*p* < 0.001). Patients that underwent surgery only (40.69%) constitute the vast majority of patients included in the study, while those treated with radiotherapy both before and after surgery (0.57%) were the least representative, especially in elderly patients. PORT with surgery constituted an increasing proportion of therapeutic procedures during the period considered (26.27% from 2004 to 2007, 31.53% from 2008 to 2011, and 42.20% from 2012 to 2017), whereas preoperative radiotherapy with surgery decreased (36.96% from 2004 to 2007, 33.13% from 2008 to 2011, and 29.91% from 2012 to 2017) in the same period (Fig. 2), which means during this period, PORT became more and more popular than preoperative radiotherapy. The majority of patients over 75 years of age refused radiotherapy (84.83%) regardless of whether they underwent surgery, while the refusal rate was only at 67.43% in patients younger than 75 years. Additionally, patients who underwent radiotherapy combined with surgery (over 90%) were more likely to receive chemotherapy than those who only had surgery (44.32%). Moreover, patients who refused radiotherapy were older (30.43% vs 2.32% over 75 years old) and less treated with chemotherapy than others (58.33% vs 91.13%). Insured, married, high median household income people were more likely to have surgery and surgery combined with radiotherapy than uninsured, unmarried, and lower median household income people.

### Univariate and multivariate analysis

In the univariate COX regression analysis of OS and LCSS, compared with patients underwent surgery only, the hazard ratio (HR), 95% confidence interval (CI) [HR(95% CI)] of patients that underwent preoperative radiotherapy was 0.477 (0.429–0.531) and 0.507 (0.452–0.568) for OS and LCSS, respectively. That of PORT patients was 0.632 (0.602–0.662) and 0.645 (0.612–0.679); of patients that underwent radiotherapy both before and after surgery was 0.593 (0.466–0.755) and 0.56 (0.426–0.736), and of patients that underwent neither radiotherapy nor surgery was 1.052 (1.010–1.095) and 1.057(1.011–1.104). All the *p*-values were less than 0.05. This analysis also showed that the following parameters are associated with a significantly shorter OS and LCSS: female sex, old age, not AD, no chemotherapy, larger tumor, higher grade, no surgery or radiotherapy, white ethnicity, earlier year of diagnosis, non-upper lobe primary lesion, higher grade, unmarried, low income.

According to the multivariate analysis, age, sex, tumor size, histology, laterality, primary site, pathologic differentiation grade, chemotherapy and radiotherapy with surgery variables were statistically significant (*p* < 0.001). The multivariate analysis showed that all the four combination of surgery and radiotherapy promoted a better survival than having neither surgery nor radiotherapy. Patients with only surgery were taken as the reference for the subsequent analysis. The HR (95% CI, p) of patients that underwent preoperative radiotherapy was 0.589 (0.529–0.657), *p* < 0.001 and 0.606 (0.539–0.681), *p* < 0.001 for OS and LCSS, respectively. For patients that underwent PORT was 0.775 (0.737–0.816), *p* < 0.001 and 0.772 (0.731–0.816), *p* < 0.001 and for patients that underwent radiotherapy both before and after surgery was 0.752 (0.590–0.957), *p* = 0.021 and 0.687 (0.522–0.904), *p* = 0.007. For patients who refused surgery or radiotherapy, *p* values were not significant. Results of the univariate and multivariate Cox regression of prognostic factors for OS and LCSS in IIIA/N2 NSCLC patients are shown in Table 2.

**Table 2:** Multivariate COX hazards regression for OS and LCSS in IIIA/N2 NSCLC patients based on prognostic factors

Variables	OS				LCSS			
	Univariate analysis		Multivariate analysis		Univariate analysis		Multivariate analysis	
	HR(95% Cl)	P	HR(95% Cl)	P	HR(95% Cl)	P	HR(95% Cl)	P
**Gender**		<0.001		<0.001		<0.001		<0.001
Female	Reference		Reference		Reference		Reference	
Male	0.799(0.772,0.828)	<0.001	0.823(0.794,0.853)	<0.001	0.823(0.792,0.855)	<0.001	0.845(0.813,0.879)	<0.001
**Age**		<0.001		<0.001		<0.001		<0.001
< 75	Reference		Reference		Reference		Reference	
> =75	1.645(1.582,1.710)	<0.001	1.364(1.310,1.421)	<0.001	1.590(1.524,1.659)	<0.001	1.334(1.276,1.395)	<0.001
**Histology**		<0.001		<0.001		<0.001		<0.001
Adenocarcinoma	Reference		Reference		Reference		Reference	
Squamous	1.429(1.372,1.489)	<0.001	1.228(1.177,1.281)	<0.001	1.388(1.328,1.451)	<0.001	1.193(1.139,1.250)	<0.001
Adenosquamous	1.159(1.018,1.319)	0.026	1.114(0.977,1.269)	0.106	1.138(0.988,1.312)	0.073	1.087(0.943,1.254)	0.25
Large cell	1.248(1.125,1.384)	<0.001	1.159(1.041,1.292)	0.007	1.303(1.167,1.456)	<0.001	1.209(1.077,1.357)	0.001
OTHER	1.395(1.328,1.464)	<0.001	1.187(1.128,1.250)	<0.001	1.411(1.339,1.488)	<0.001	1.196(1.132,1.265)	<0.001
**Chemotherapy**		<0.001		<0.001		<0.001		<0.001
No	Reference		Reference		Reference		Reference	
Yes	0.556(0.536,0.577)	<0.001	0.635(0.610,0.662)	<0.001	0.586(0.563,0.610)	<0.001	0.665(0.636,0.695)	<0.001
**Tumor Size**		<0.001		<0.001		<0.001		<0.001
≤ 3	Reference		Reference		Reference		Reference	
3--5	1.275(1.231,1.320)	<0.001	1.221(1.178,1.266)	<0.001	1.311(1.261,1.362)	<0.001	1.258(1.209,1.308)	<0.001
**Grade**		<0.001		<0.001		<0.001		<0.001
Grade I	Reference		Reference		Reference		Reference	
Grade II	1.128(1.015,1.253)	0.025	1.134(1.020,1.260)	0.02	1.147(1.021,1.289)	0.021	1.154(1.027,1.297)	0.016
Grade III	1.34(1.210,1.485)	<0.001	1.334(1.203,1.480)	<0.001	1.399(1.249,1.567)	<0.001	1.386(1.235,1.554)	<0.001
Grade IV	1.593(1.351,1.880)	<0.001	1.512(1.275,1.794)	<0.001	1.604(1.336,1.926)	<0.001	1.495(1.237,1.806)	<0.001
unknow	1.569(1.417,1.737)	<0.001	1.496(1.348,1.660)	<0.001	1.627(1.453,1.821)	<0.001	1.544(1.377,1.732)	<0.001
**Radiation with surgery**		<0.001		<0.001		<0.001		<0.001
Only surgery	Reference		Reference		Reference		Reference	
Preoperative radiotherapy	0.477(0.429,0.531)	<0.001	0.589(0.529,0.657)	<0.001	0.507(0.452,0.568)	<0.001	0.606(0.539,0.681)	<0.001
postoperative radiotherapy	0.632(0.602,0.662)	<0.001	0.775(0.737,0.816)	<0.001	0.645(0.612,0.679)	<0.001	0.772(0.731,0.816)	<0.001
Raiotherapy both before and after sugery	0.593(0.466,0.755)	<0.001	0.752(0.590,0.957)	0.021	0.56(0.426,0.736)	<0.001	0.687(0.522,0.904)	0.007
No surgery or radiation	1.052(1.010,1.095)	0.015	1.035(0.991,1.082)	0.121	1.057(1.011,1.104)	0.014	1.023(0.975,1.073)	0.356
**Primary Site**		<0.001		<0.001		<0.001		<0.001
Upper lobe	Reference		Reference		Reference		Reference	
Middle lobe	1.026(0.943,1.116)	0.557	1.009(0.926,1.099)	0.833	1.053(0.962,1.153)	0.26	1.026(0.935,1.124)	0.592
Lower lobe	1.103(1.059,1.149)	<0.001	1.08(1.037,1.125)	<0.001	1.102(1.054,1.151)	<0.001	1.078(1.032,1.127)	0.001
NOS	1.303(1.172,1.449)	<0.001	1.314(1.172,1.474)	<0.001	1.29(1.148,1.449)	<0.001	1.311(1.157,1.486)	<0.001
Overlapping lesion	0.985(0.808,1.199)	0.877	1.024(0.840,1.248)	0.814	0.922(0.738,1.153)	0.478	0.945(0.756,1.182)	0.62
Main bronchus	1.252(1.136,1.379)	<0.001	1.128(1.023,1.245)	0.016	1.302(1.174,1.444)	<0.001	1.171(1.054,1.301)	0.003
**Laterality**		0.001		<0.001		<0.001		<0.001
Right-origin of primary	Reference		Reference		Reference		Reference	
Left-origin of primary	0.932(0.899,0.966)	<0.001	0.926(0.893,0.961)	<0.001	0.907(0.872,0.944)	<0.001	0.904(0.868,0.941)	<0.001
Paired sit	0.836(0.633,1.104)	0.206	0.717(0.532,0.966)	0.029	0.809(0.595,1.100)	0.177	0.694(0.500,0.964)	0.029
Only one side - side unspecified	1.556(0.861,2.810)	0.143	1.339(0.737,2.430)	0.338	1.14(0.543,2.391)	0.73	0.991(0.470,2.090)	0.982
Not a paired site	1.266(0.408,3.927)	0.683	1.371(0.438,4.288)	0.587	1.532(0.494,4.752)	0.46	1.647(0.526,5.158)	0.392

Abbreviations: *NSCLC* Non-small cell lung cancer, *IIIA/N2* Stage IIIA pathologic N2, *OS* Overall survival, *LCSS* Lung cancer-specific survival, *HRs* Hazard ratios

### Survival outcomes

The median follow-up for the whole cohort was 39 months for OS and 48 months for LCSS. The median follow-up for the surgery only, preoperative radiotherapy with surgery, PORT with surgery, radiotherapy both before and after surgery, and no surgery or radiotherapy groups were 36, 66, 51, 55 and 31 months, respectively for OS and 45, 72, 59, 66 and 40 months for LCSS. Patients who received preoperative radiotherapy with surgery had the longest 5-year overall survival (42.84%) and lung cancer-specific survival (47.12%).

**Fig. 3:**
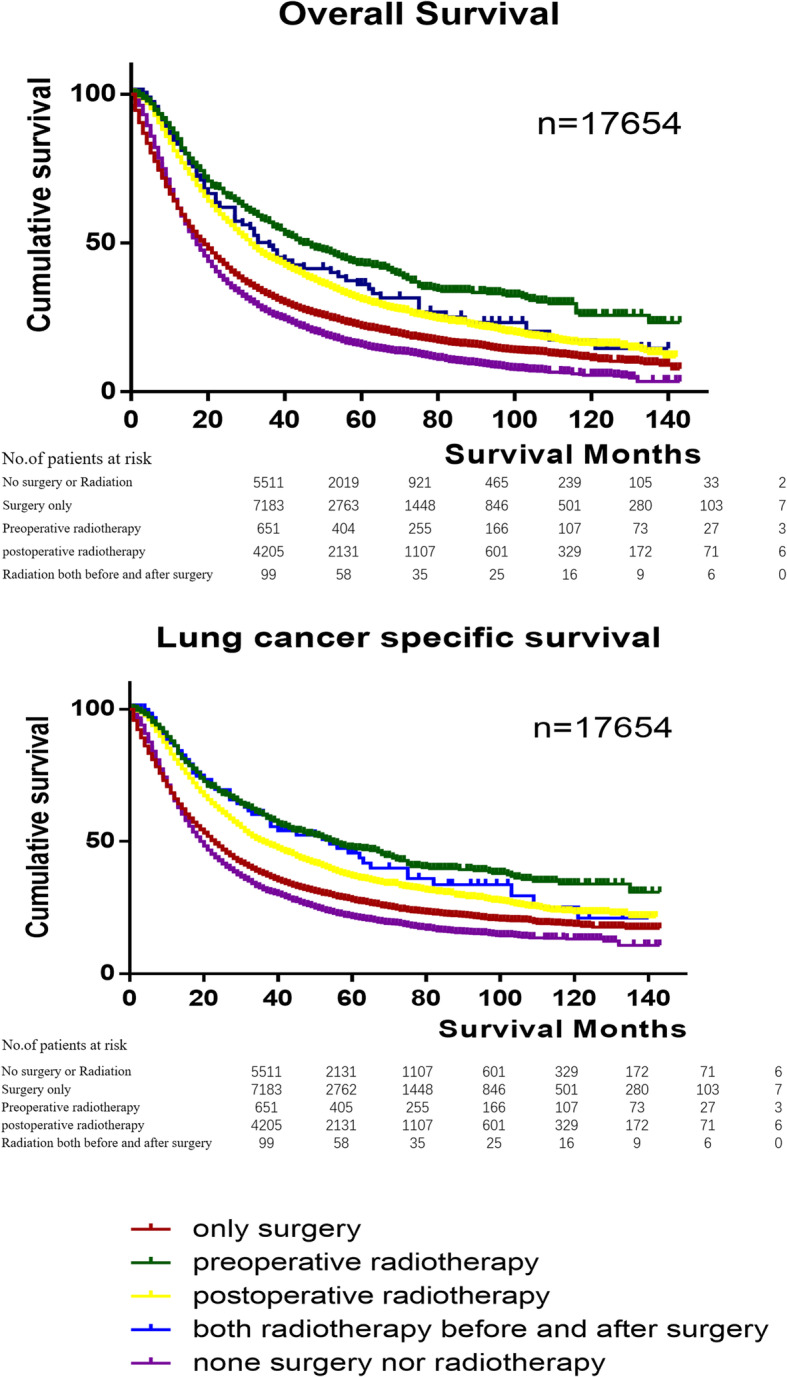
Kaplan–Meier analysis of different radiotherapy sequences on OS and LCSS of IIIA/N2 NSCLC patients. It showed that both in OS and LCSS of IIIA/N2 NSCLC patients, preoperative radiotherapy was the best strategy, and then were both preoperative and PORT, PORT, only surgery and neither surgery nor radiotherapy. Log Rank *p* < 0.001. The number of patients at risk in different time periods was under survival curves. Abbreviations: NSCLC, non–small cell lung cancer; IIIA/N2, stage IIIA pathologic N2; OS, Overall survival; LCSS, Lung cancer specific survival

The Kaplan-Meier method was used to estimate the OS and LCSS, showing that preoperative radiotherapy was the optimal strategy among IIIA/N2 patients (*p* < 0.001). Moreover, patients that underwent surgery had better survival than who refused it (*p* < 0.001). The survival of patients that underwent surgery combined with radiotherapy was better than patients who underwent surgery only (*p* < 0.001). The Survival analysis was performed using the log-rank test, and showed that the pairwise difference between each groups were statistically significant (Fig. 3).

**Fig. 4:**
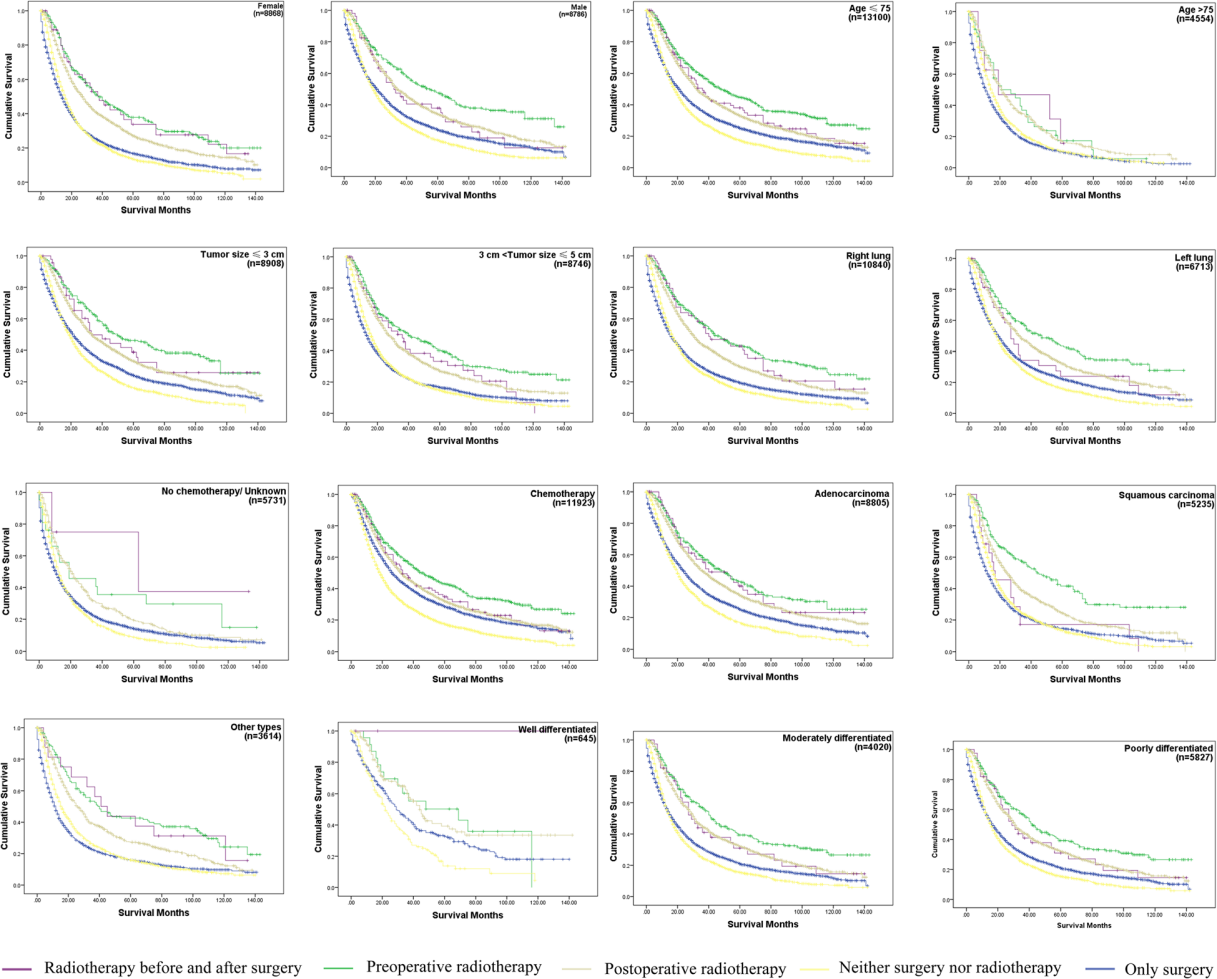
The OS curves in IIIA/N2 NSCLC subgroups with different surgery and radiotherapy combination. Log Rank *p* < 0.001 each subgroup. In subgroup age > 75, p- value of the OS of patients treated by preoperative radiotherapy compared to patients who underwent PORT was 0.6848. Each *p*-value < 0.05 in the pairwise comparison of other subgroups. Abbreviations: NSCLC, non-small cell lung cancer; IIIA/N2, stage IIIA pathologic N2; OS, Overall survival; PORT, postoperative radiotherapy. The number of patients at risk in different time periods was in Supplementary Table 1

**Fig. 5:**
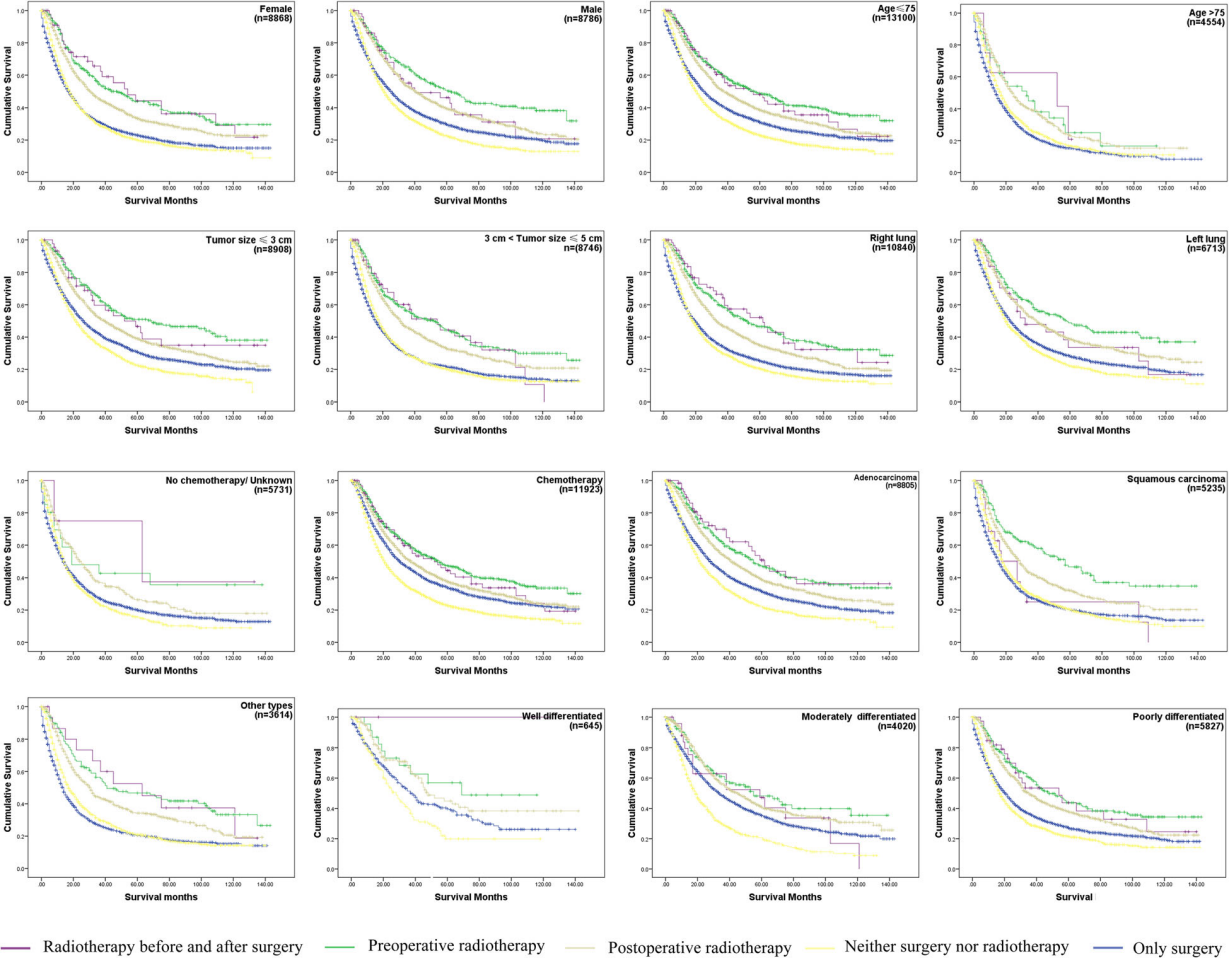
The LCSS curves in IIIA/N2 NSCLC subgroups with different surgery and radiotherapy combination. Log Rank *p* < 0.001 in each subgroup. p-value of the lung cancer-specific survival of patients treated by preoperative radiotherapy compared to patients who underwent PORT was 0.5293. Each *p* value < 0.05 in the pairwise comparison of other subgroups. The number of patients at risk in different time periods was in Supplementary Table 1. Abbreviations: NSCLC, non-small cell lung cancer; IIIA/N2, stage IIIA pathologic N2; LCSS, Lung cancer-specific survival; PORT, postoperative radiotherapy

Similar results were observed in the subgroups (sex, age, tumor size, primary site, chemotherapy, histology, pathologic differentiation grade) analysis of the OS and LCSS Kaplan-Meier. Importantly, considering that most patients with IIIA/N2 NSCLC received chemotherapy in clinical, the chemotherapy subgroup was the most practical one. In chemotherapy subgroup, there were almost the same survival curves as overall survival curves and the survival rate of preoperative radiotherapy of both OS and LCSS were the highest in this subgroup. Interestingly, we also found that the optimal treatment for the subgroup of patients with > 75 years old was PORT, while in the subgroup of no chemotherapy the optimal treatment may be radiotherapy both before and after surgery (Fig. 4, Fig. 5). The OS and LCSS analysis showed that the survival rate of patients that underwent preoperative radiotherapy was not significantly different with patients who underwent PORT in the AD subgroup (*p* = 0.8274 and 0.7653 for OS and LCSS analysis, respectively). Moreover, there was no significant difference between the survival of patients who refused surgery and that of patients who received surgery only (*p* = 0.6848 and 0.5293 for OS and LCSS analysis, respectively) in the subgroup of patients with age > 75. Thus, the quality of life may be the main consideration for IIIA/N2 NSCLC patients with age > 75.

## Discussion

In this study, we used the SEER Database to analyze the prognostic value of surgery and radiotherapy combinations and the relationship between therapeutic strategy with survival and HRs in IIIA/N2 NSCLC patients. Our data demonstrated that the use of surgery in IIIA/N2 NSCLC patients was effective in all the analyzed groups, except in patients older than 75 years old. In subgroup of age > 75, surgery did not improve survival, and PORT was more recommended if having surgery and radiation. For patients who underwent surgery combined with radiotherapy, the preoperative radiotherapy regimen led to the best result, while those who underwent neither surgery nor radiotherapy had the worst prognosis. Clinically, since adjuvant chemotherapy has become a standard treatment, the results of the chemotherapy subgroup deserve special attention, which were generally consistent with the overall OS and LCSS. The LCSS rate in the overall patients was similar to of the OS rate. In the Cox-regression analysis, the following parameters were associated with a higher risk of death: female sex, age > 75, SCC and other histologic types, no chemotherapy, poor differentiation, and larger tumor. With so many high-risk parameters, the results of survival analysis in subgroups were of great significance and we will further analyze the influence of age, gender, histologic types, chemotherapy, pathologic differentiation grade and tumor size on the survival of IIIA/N2 NSCLC patients in the future.

There was significant heterogeneity in the survival rate of IIIA/N2 NSCLC patients, for which the standard treatment had been debated for a long time [11, 21–23]. Although the optimal treatment approach in IIIA/N2 NSCLC patients remains undetermined, almost all studies confirmed the effectiveness of surgery. A recent review argued that in patients with IIIA/N2 NSCLC, radical resection with lymph node dissection is reasonable and early operation leads to a greater benefit [24]. Bryan DS et al. found that surgery improve survival in IIIA/N2 NSCLC, and in a survey, most thoracic surgeons recommended surgery as part of the therapy for IIIA/N2 NSCLC patients, because resection improves the rate of local control [25]. Consistently with this view, patients younger than 75 years old who underwent surgery showed an improved survival rate compared to those that do not in our study. However, there is still a lack of high-quality prospective evidence showing that how the different surgical procedures influence the survival rates.

Historically, surgery alone or combined with chemotherapy and radiotherapy has been the most common approach, and in recent decades there is a trend of performing radiotherapy before surgery. Previous studies reported that radiotherapy followed by surgery gave a survival benefit for IIIA/N2 NSCLC patients [26–29]. Several trials have been performed to determine the safety and efficacy of the combination of chemo- and radiotherapy [13, 27, 30–34]. Some of those reports demonstrated that chemotherapy combined with radiotherapy prolonged the survival of III/N2 NSCLC patients, as we found in this study [27, 30] while one clinical trial showed that radiotherapy combined with chemotherapy does not improve survival of IIIA/N2 NSCLC patients that underwent surgery [33]. Another study [35] suggested that surgery combined with chemoradiotherapy could improve the patients’ survival. Therefore, in previous studies there is no consensus for the optimal combination time of radiotherapy and chemotherapy [36]. In our study, we demonstrated that preoperative radiotherapy is the best strategy for approaches combining chemo and radiotherapy, consistently with a previous study based on SEER data [17], as well as another independent study [13].

The subgroup of patients with an age > 75 was different from the others, because surgery only did not appear to significantly improve survival in comparison to no surgery. PORT was the optimal treatment strategy in cases having surgery. These results can be explained by a worse treatment tolerance in this subgroup. In addition to that, these patients also underwent radiotherapy less frequently and had a higher death risk, consistently to a previous study [37]. Therefore, it is important to determine optimal treatment for the elderly population. However, older and frail patients are often excluded from clinical trials using strict eligibility criteria [38] and receive less standard treatment [39, 40]. Thus, future research should focus on the elderly population who could benefit from specific treatment regimens.

The SEER database includes a population size larger than other clinical trials [41], and the inclusion of the revision made by the TNM classification project makes it more reliable to predict the survival outcomes [42–44]. This study has several limitations: other than the intrinsic defects of retrospective study, important information, such as gene mutations, lymph nodes station, chemotherapy sequences, and extent of resection are not provided in the SEER database. Moreover, patients treated with radiotherapy are more likely to be treated with chemotherapy as well. As role of chemotherapy must be seriously taken into account [30], this study could have a bias favoring radiotherapy. Future prospective studies will need more detailed information on chemotherapy data.

## Conclusions

According to this study, the different clinical outcomes between different strategies of surgery and radiotherapy combination may be useful to determine prognosis and to provide an important reference for clinicians and patients. Our data revealed that surgery, radiotherapy and chemotherapy were associated with improved OS and LCSS in patients with IIIA/N2 NSCLC. Younger patients underwent surgical resection and preoperative radiotherapy had the best chance of survival (*p* < 0.001). Based on our results, the rank of therapeutic strategies to improve OS and LCSS in IIIA/N2 NSCLC patients is the following: preoperative radiotherapy > radiotherapy both before and after surgery (considering the small number of people in this subgroup, we do not make a positive recommendation for this sequence, but this method should not be rejected or excluded, it worth more detailed relevant research in the future) > PORT > only surgery > neither surgery nor radiotherapy (*p* < 0.001). In conclusion, preoperative radiotherapy is worth attention. However, in patients with more than 75 years, there was no clear benefit from surgery. The quality of life should be the main consideration for them when making choice. Novel treatments like immunotherapy and targeting therapy as additional options will need to be evaluated in future studies.

## Supplementary information

**Additional file 1: Table S1.** The number of patients at risk in different time periods in different subgroups.

## Data Availability

All the data in the article was from SEER database at http://seer.cancer.gov/., which contain data from 18 registered cancer institute covering nearly 26% of the total US population.

## Abbreviations

NSCLC: Non–small cell lung cancer
IIIA/N2: Stage IIIA pathologic N2
OS: Overall survival
LCSS: Lung cancer-specific survival
SEER: Surveillance, Epidemiology, and End Results registry
HRs: Hazard ratios
PORT: Postoperative radiotherapy
AD: Adenocarcinoma
SQCC: Squamous cell carcinoma
LCC: Large cell carcinoma
