# Supplementary material for: The impact of order with radiation therapy in stage IIIA pathologic N2 NSCLC patients: a population-based study
Source: BMC Cancer. 2020 Aug 26;20:809. doi: 10.1186/s12885-020-07309-y (PMC7448510; doi:10.1186/s12885-020-07309-y)
Supplement: Supplementary file 1 — Additional file 1: Table S1. The number of patients at risk in different time periods in different subgroups. [file 12885_2020_7309_MOESM1_ESM.doc]

**Supplementary Table 1. The number of patients at risk in different** **time periods in different subgroups.**

No.of female patients at risk

| No surgery or Radiation | 2939 | 997 | 441 | 215 | 118 | 53 | 17 | 1 |
| --- | --- | --- | --- | --- | --- | --- | --- | --- |
| Surgery only | 3503 | 1173 | 595 | 345 | 208 | 120 | 43 | 4 |
| Preoperative radiotherapy | 322 | 198 | 114 | 72 | 50 | 32 | 9 | 1 |
| postoperative radiotherapy | 2053 | 954 | 487 | 257 | 141 | 73 | 35 | 1 |
| Radiation both before and after surgery | 46 | 27 | 18 | 11 | 8 | 4 | 3 | 0 |

No.of male patients at risk

| No surgery or Radiation | 2571 | 1022 | 480 | 250 | 121 | 52 | 16 | 1 |
| --- | --- | --- | --- | --- | --- | --- | --- | --- |
| Surgery only | 3679 | 1590 | 853 | 501 | 293 | 160 | 60 | 3 |
| Preoperative radiotherapy | 328 | 207 | 141 | 94 | 57 | 41 | 18 | 1 |
| postoperative radiotherapy | 2151 | 1177 | 620 | 344 | 188 | 99 | 36 | 5 |
| Radiation both before and after surgery | 52 | 31 | 17 | 14 | 8 | 4 | 2 | 0 |

No.of age≤75 patients at risk

| No surgery or Radiation | 3193 | 1090 | 485 | 253 | 133 | 55 | 17 | 2 |
| --- | --- | --- | --- | --- | --- | --- | --- | --- |
| Surgery only | 4939 | 2150 | 1204 | 722 | 435 | 262 | 93 | 6 |
| Preoperative radiotherapy | 607 | 384 | 244 | 161 | 106 | 72 | 28 | 3 |
| postoperative radiotherapy | 3566 | 1878 | 991 | 561 | 308 | 162 | 65 | 6 |
| Radiation both before and after surgery | 52 | 28 | 17 | 13 | 8 | 3 | 1 | 0 |

No.of age>75 patients at risk

| No surgery or Radiation | 1618 | 501 | 201 | 96 | 49 | 22 | 4 | 0 |
| --- | --- | --- | --- | --- | --- | --- | --- | --- |
| Surgery only | 2243 | 613 | 244 | 124 | 66 | 27 | 11 | 1 |
| Preoperative radiotherapy | 43 | 21 | 11 | 5 | 2 | 0 | 0 | 0 |
| postoperative radiotherapy | 638 | 253 | 116 | 40 | 21 | 10 | 6 | 0 |
| Radiation both before and after surgery | 7 | 3 | 1 | 0 | 0 | 0 | 0 | 0 |

No.of tumor size≤3cm patients at risk

| No surgery or Radiation | 2317 | 929 | 436 | 212 | 106 | 50 | 16 | 0 |
| --- | --- | --- | --- | --- | --- | --- | --- | --- |
| Surgery only | 3764 | 1647 | 902 | 516 | 316 | 178 | 70 | 6 |
| Preoperative radiotherapy | 294 | 198 | 127 | 82 | 57 | 34 | 11 | 0 |
| postoperative radiotherapy | 2482 | 1293 | 703 | 400 | 220 | 118 | 50 | 5 |
| Radiation both before and after surgery | 46 | 30 | 18 | 12 | 8 | 6 | 5 | 0 |

No.of 3cm<tumor size≤5cm patients at risk

| No surgery or Radiation | 3193 | 1090 | 485 | 253 | 133 | 55 | 18 | 2 |
| --- | --- | --- | --- | --- | --- | --- | --- | --- |
| Surgery only | 3418 | 1116 | 546 | 330 | 185 | 102 | 33 | 2 |
| Preoperative radiotherapy | 356 | 207 | 128 | 84 | 51 | 39 | 16 | 2 |
| postoperative radiotherapy | 1722 | 838 | 404 | 201 | 109 | 54 | 23 | 1 |
| Radiation both before and after surgery | 52 | 28 | 17 | 13 | 8 | 3 | 1 | 0 |

No.of origin right lung patients at risk

| No surgery or Radiation | 3420 | 1241 | 575 | 283 | 147 | 64 | 23 | 1 |
| --- | --- | --- | --- | --- | --- | --- | --- | --- |
| Surgery only | 4315 | 1593 | 841 | 482 | 297 | 166 | 64 | 4 |
| Preoperative radiotherapy | 404 | 251 | 162 | 101 | 66 | 47 | 18 | 3 |
| postoperative radiotherapy | 2630 | 1314 | 667 | 356 | 186 | 93 | 41 | 5 |
| Radiation both before and after surgery | 66 | 38 | 26 | 20 | 9 | 5 | 4 | 0 |

No.of origin left lung patients at risk

| No surgery or Radiation | 2062 | 769 | 342 | 180 | 90 | 39 | 11 | 2 |
| --- | --- | --- | --- | --- | --- | --- | --- | --- |
| Surgery only | 2839 | 1163 | 604 | 362 | 203 | 113 | 38 | 3 |
| Preoperative radiotherapy | 245 | 154 | 93 | 65 | 41 | 27 | 9 | 0 |
| postoperative radiotherapy | 1530 | 792 | 429 | 237 | 137 | 77 | 30 | 1 |
| Radiation both before and after surgery | 32 | 20 | 10 | 8 | 7 | 4 | 2 | 0 |

No.of no chemotherapy patients at risk

| No surgery or Radiation | 1290 | 373 | 153 | 70 | 27 | 11 | 3 | 0 |
| --- | --- | --- | --- | --- | --- | --- | --- | --- |
| Surgery only | 3999 | 1179 | 576 | 343 | 210 | 117 | 47 | 6 |
| Preoperative radiotherapy | 20 | 9 | 7 | 6 | 5 | 4 | 2 | 0 |
| postoperative radiotherapy | 414 | 173 | 80 | 42 | 24 | 9 | 6 | 1 |
| Radiation both before and after surgery | 3 | 2 | 2 | 2 | 1 | 1 | 1 | 0 |

No.of chemotherapy patients at risk

| No surgery or Radiation | 4220 | 1646 | 768 | 395 | 212 | 94 | 31 | 2 |
| --- | --- | --- | --- | --- | --- | --- | --- | --- |
| Surgery only | 3183 | 1584 | 872 | 503 | 291 | 163 | 56 | 3 |
| Preoperative radiotherapy | 630 | 396 | 248 | 160 | 102 | 69 | 25 | 3 |
| postoperative radiotherapy | 3790 | 1958 | 1027 | 559 | 305 | 163 | 65 | 5 |
| Radiation both before and after surgery | 95 | 56 | 33 | 23 | 15 | 8 | 5 | 0 |

No.of adenocarcinoma patients at risk

| No surgery or Radiation | 2080 | 812 | 362 | 172 | 84 | 32 | 13 | 1 |
| --- | --- | --- | --- | --- | --- | --- | --- | --- |
| Surgery only | 3830 | 1729 | 918 | 520 | 302 | 159 | 62 | 5 |
| Preoperative radiotherapy | 341 | 214 | 132 | 77 | 42 | 29 | 13 | 1 |
| postoperative radiotherapy | 2485 | 1320 | 700 | 371 | 197 | 105 | 43 | 6 |
| Radiation both before and after surgery | 64 | 38 | 24 | 16 | 10 | 5 | 4 | 0 |

No.of squamous patients at risk

| No surgery or Radiation | 2117 | 697 | 290 | 135 | 64 | 22 | 5 | 1 |
| --- | --- | --- | --- | --- | --- | --- | --- | --- |
| Surgery only | 1921 | 595 | 290 | 168 | 102 | 71 | 22 | 1 |
| Preoperative radiotherapy | 172 | 102 | 64 | 41 | 24 | 16 | 6 | 0 |
| postoperative radiotherapy | 1002 | 442 | 198 | 99 | 55 | 26 | 13 | 0 |
| Radiation both before and after surgery | 18 | 8 | 2 | 2 | 2 | 2 | 1 | 0 |

No.of other type patients at risk

| No surgery or Radiation | 1312 | 510 | 269 | 158 | 91 | 51 | 15 | 0 |
| --- | --- | --- | --- | --- | --- | --- | --- | --- |
| Surgery only | 1430 | 439 | 240 | 158 | 97 | 50 | 19 | 1 |
| Preoperative radiotherapy | 136 | 89 | 59 | 48 | 41 | 28 | 8 | 2 |
| postoperative radiotherapy | 716 | 369 | 209 | 131 | 77 | 41 | 15 | 0 |
| Radiation both before and after surgery | 15 | 12 | 9 | 7 | 4 | 2 | 1 | 0 |

No.of well differentiated patients at risk

| No surgery or Radiation | 153 | 64 | 28 | 9 | 4 | 2 | 0 | 0 |
| --- | --- | --- | --- | --- | --- | --- | --- | --- |
| Surgery only | 326 | 179 | 102 | 63 | 36 | 15 | 5 | 0 |
| Preoperative radiotherapy | 22 | 17 | 11 | 9 | 4 | 2 | 0 | 0 |
| postoperative radiotherapy | 137 | 74 | 41 | 23 | 13 | 8 | 5 | 1 |
| Radiation both before and after surgery | 2 | 1 | 0 | 0 | 0 | 0 | 0 | 0 |

No.of moderately differentiated patients at risk

| No surgery or Radiation | 899 | 311 | 117 | 63 | 32 | 11 | 4 | 0 |
| --- | --- | --- | --- | --- | --- | --- | --- | --- |
| Surgery only | 1934 | 961 | 536 | 313 | 174 | 98 | 38 | 3 |
| Preoperative radiotherapy | 137 | 84 | 50 | 29 | 16 | 12 | 6 | 0 |
| postoperative radiotherapy | 1020 | 595 | 329 | 172 | 91 | 50 | 22 | 2 |
| Radiation both before and after surgery | 25 | 14 | 10 | 8 | 4 | 3 | 1 | 0 |

No.of poorly differentiated patients at risk

| No surgery or Radiation | 1721 | 594 | 280 | 147 | 75 | 35 | 15 | 1 |
| --- | --- | --- | --- | --- | --- | --- | --- | --- |
| Surgery only | 2426 | 951 | 519 | 312 | 194 | 115 | 40 | 3 |
| Preoperative radiotherapy | 277 | 176 | 110 | 70 | 51 | 35 | 13 | 3 |
| postoperative radiotherapy | 1360 | 718 | 378 | 224 | 121 | 65 | 24 | 3 |
| Radiation both before and after surgery | 38 | 26 | 13 | 8 | 7 | 4 | 3 | 0 |
